# Bundeslandübergreifende Förderung der Impfprävention in Deutschland mit Orientierung an europäischen Zielen: die Nationale Lenkungsgruppe Impfen (NaLI)

**DOI:** 10.1007/s00103-025-04026-4

**Published:** 2025-03-20

**Authors:** Jens Milbradt, Maria-Sabine Ludwig

**Affiliations:** Geschäftsstelle der Nationalen Lenkungsgruppe Impfen (NaLI), am Bayerischen Landesamt für Gesundheit und Lebensmittelsicherheit (LGL), Eggenreuther Weg 43, 91058 Erlangen, Deutschland

**Keywords:** Impfprävention, Nationale Lenkungsgruppe Impfen (NaLI), Nationaler Impfplan, European Immunization Agenda 2030 (EIA2030), Impfziele, Vaccination, National Immunization Steering Group (NaLI), National Vaccination Plan, European Immunization Agenda 2030 (EIA2030), Immunization targets

## Abstract

Die Nationale Lenkungsgruppe Impfen (NaLI), 2016 gegründet, ist ein Bund-Länder-Gremium, das den länderübergreifenden Austausch zu Impfstrategien fördert und die Entwicklung gemeinsamer Konzepte zur Verbesserung der Impfprävention vorantreibt. Eine der Hauptaufgaben der NaLI ist die Umsetzung, Erfolgskontrolle und Fortschreibung des Nationalen Impfplans von 2012. Dieser Plan informiert über die komplexe Organisation des föderalen Impfwesens in Deutschland und formuliert Ziele sowie Maßnahmen, um den Impfschutz der Bevölkerung zu verbessern.

Neben der Bestandsaufnahme der Umsetzung des Nationalen Impfplans beschreibt dieser Beitrag die bisherigen Anstrengungen zur Ergänzung und Weiterentwicklung des Plans. Dazu gehören die transparente Darstellung des deutschen Impfwesens auf der NaLI-Website, der 2015 veröffentlichte „Nationale Aktionsplan zur Elimination der Masern und Röteln“ sowie das derzeit erarbeitete NaLI-Konzept zur HPV-Impfung.

In den letzten Jahren wurden einige der Ziele des Nationalen Impfplans bereits erreicht und viele Aspekte haben sich weiterentwickelt. Daher strebt die NaLI aktuell eine Überarbeitung des Plans an, bei der Erfahrungen aus der bisherigen Umsetzung sowie neue wissenschaftliche Erkenntnisse einfließen sollen. Internationale Strategien und Zielvorgaben, insbesondere die „European Immunization Agenda 2030“, dienen dabei als wichtige Orientierung. Die Agenda formuliert Ziele, die über das Erreichen definierter Impfquoten hinausgehen. Im Vordergrund stehen ein gleichberechtigter Zugang zu Impfungen, das Konzept des lebensbegleitenden Impfens und die Ausbruchskontrolle impfpräventabler Erkrankungen. Die Anpassung und Aktualisierung des Nationalen Impfplans verfolgen das Ziel, eine bundesweit koordinierte Förderung der Impfprävention zu erreichen.

## Abstract (en)

The National Immunization Steering Group (NaLI), founded in 2016, is a federal-state committee that promotes the exchange of information on immunization strategies across state borders and encourages the development of joint approaches. One of the main tasks of the NaLI is to implement, monitor, and update the National Vaccination Plan of 2012, which provides information on the complex organization of the federal vaccination system in Germany and formulates goals and measures to improve the vaccination protection of the population.

In addition to a brief evaluation of the implementation of the National Vaccination Plan of 2012, this article describes the efforts made to date to supplement and further develop the plan. In particular, these include the transparent presentation of the German vaccination system on the NaLI website, the National Action Plan for the Elimination of Measles and Rubella published in 2015, and a concept for HPV vaccination currently being developed by the NaLI.

In recent years, some of the goals of the National Vaccination Plan have been achieved and many aspects have evolved. The NaLI therefore intends to revise the plan, taking into account the experience gained during its implementation and new scientific evidence. International policies and targets, in particular the European Immunization Agenda 2030, provide important guidance. The agenda sets targets that go beyond achieving specific immunization coverage levels. It focuses on equitable access to immunization, the concept of immunization delivery across the life course, and the control of outbreaks of vaccine-preventable diseases. The aim of the adaptation and update of the National Vaccination Plan is to achieve a coordinated promotion of vaccination prevention throughout the country.

## Einführung

Impfprävention bietet die Chance, sowohl die individuelle als auch die öffentliche Gesundheit zu stärken. Impfungen können vor schweren Krankheiten schützen, Hospitalisierungen verringern und die Sterblichkeit senken. Bei vielen Impfungen – beispielsweise gegen Masern, Diphtherie und humane Papillomviren – kann eine hohe Impfquote den Gemeinschaftsschutz fördern und so auch gefährdete Personengruppen besser schützen. Zudem trägt die Vermeidung von Krankheitsausbrüchen zu Kosteneinsparungen im Gesundheitssystem bei und steigert die Lebensqualität, da gesunde Menschen aktiver am sozialen Leben teilnehmen können. Insgesamt leistet die Impfprävention einen entscheidenden Beitrag zur Bekämpfung von Infektionskrankheiten weltweit.

Das Impfwesen in Deutschland ist durch eine komplexe föderale und nationale Struktur geprägt. In der vielschichtigen Organisation des Impfens agieren Bund, Länder und Kommunen eng zusammen. Der Bund legt unter anderem mit dem Infektionsschutzgesetz die rechtlichen Rahmenbedingungen fest [1]. Die Umsetzung obliegt jedoch den Ländern, die spezifische Regelungen und Empfehlungen erlassen können, was zu regionalen Unterschieden führt. Kommunale Gesundheitsämter spielen eine wichtige Rolle, indem sie Impfaktionen organisieren und die Bevölkerung informieren. Aufklärungskampagnen zur Sensibilisierung der Bevölkerung finden vor allem auf Landes- und kommunaler Ebene statt. Insgesamt ermöglicht dieser föderalistische Ansatz eine flexible und bedarfsgerechte Impfprävention in Deutschland.

Auf der anderen Seite erfordert die föderale Struktur jedoch einen erhöhten Abstimmungsbedarf, um gemeinsame Ziele zu erreichen und Maßnahmen effizient umzusetzen. Seit 2009 bieten nationale Impfkonferenzen ein Forum für diesen wichtigen überregionalen Dialog zwischen den Impfakteuren. Im Anschluss an die 1. Nationale Impfkonferenz beschloss die Gesundheitsministerkonferenz (GMK) die Entwicklung des „Nationalen Impfplans“, um das Impfwesen in Deutschland zu stärken. Wichtiger Bestandteil des 2012 veröffentlichten Nationalen Impfplans waren gemeinsame Ziele für das Impfwesen in Deutschland und zu einzelnen impfpräventablen Erkrankungen [2, 3]. Mit der Gründung der „Nationalen Lenkungsgruppe Impfen“ (NaLI) im Jahr 2016 wurde ein zentrales Element des Nationalen Impfplans geschaffen, das die länderübergreifende kontinuierliche Abstimmung von Impfthemen ermöglicht. Als nationales Bund-Länder-Gremium übernimmt die NaLI die Koordination der Umsetzung, Evaluation und Weiterentwicklung des Nationalen Impfplans.

In den folgenden Kapiteln werden die Aufgaben der NaLI sowie eine Bestandsaufnahme der Umsetzung des Nationalen Impfplans dargestellt. Zudem werden die bisherigen und laufenden Anstrengungen beschrieben, den Nationalen Impfplan im Hinblick auf neue Impfstrategien, Weiterentwicklungen im föderalen Impfwesen und die Einbeziehung internationaler Ziele zu aktualisieren und fortzuschreiben.

## Förderung der Impfprävention durch die Nationale Lenkungsgruppe Impfen

Auf Beschluss der Gesundheitsministerkonferenz (GMK) wurde die Nationale Lenkungsgruppe Impfen (NaLI) 2016 ins Leben gerufen. Mitglieder der NaLI sind die Gesundheitsministerien der 16 Bundesländer, das Bundesministerium für Gesundheit (BMG), die Kassenärztliche Bundesvereinigung (KBV), die Bundesärztekammer (BÄK) und der Verband der Privaten Krankenversicherungen. Als ständige Gäste sind zudem das Bundesinstitut für Öffentliche Gesundheit (BIÖG; ehemals Bundeszentrale für gesundheitliche Aufklärung (BZgA)), das Paul-Ehrlich-Institut (PEI), das Robert Koch-Institut (RKI), die Ständige Impfkommission (STIKO) sowie der Gemeinsame Bundesausschuss (G-BA) und der Spitzenverband Bund der Krankenkassen (GKV-Spitzenverband) in der NaLI vertreten. Den Vorsitz übernimmt jeweils das Bundesland, das die nächste Nationale Impfkonferenz ausrichtet.

Zur administrativen und koordinativen Unterstützung des jeweils amtierenden NaLI-Vorsitzes sowie der gesamten NaLI wurde parallel zur Gründung des Gremiums – gemäß den Beschlüssen der GMK – die Geschäftsstelle der NaLI am Bayerischen Landesamt für Gesundheit und Lebensmittelsicherheit (LGL) eingerichtet [4].

**Abb. 1:**
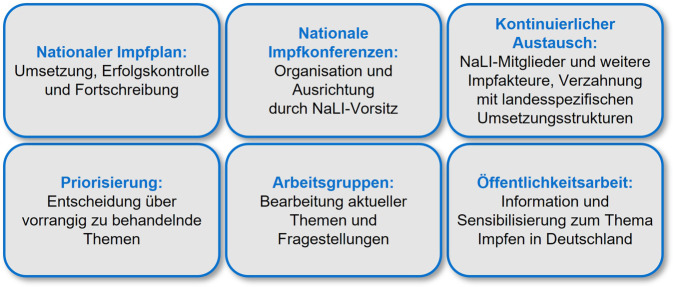
Aufgabenfelder der „Nationalen Lenkungsgruppe Impfen“ (NaLI) in Anlehnung an Beschlüsse der Gesundheitsministerkonferenz und der Arbeitsgruppe der Obersten Landesgesundheitsbehörden. (Quelle: Eigene Abbildung)

Die Aufgaben und Ziele der NaLI sind vielfältig (Abb. 1) und bedürfen einer engen Zusammenarbeit aller Mitglieder und Beteiligten. Im Zentrum stehen die Fortschreibung des Nationalen Impfplans, die Koordination der Umsetzung der im Nationalen Impfplan genannten Ziele auf nationaler und regionaler Ebene sowie eine kontinuierliche Erfolgskontrolle der umgesetzten Maßnahmen. Dieser Prozess erfolgt im Dialog mit den jeweils verantwortlichen Bundes- und Landesbehörden und anderen relevanten Akteuren und umfasst sämtliche im Nationalen Impfplan genannten Aufgabenschwerpunkte.

Um die gesteckten Ziele zu erreichen, entscheidet die NaLI über vorrangige Themen, verabschiedet Vorschläge und beschließt Empfehlungen zu Impfzielen sowie zum Nationalen Impfplan. Sie unterstützt das ausrichtende Land der Nationalen Impfkonferenz bei der thematischen und strategischen Schwerpunktsetzung und stellt die kontinuierliche fachliche Verzahnung mit den spezifischen Umsetzungsstrukturen des Nationalen Impfplans auf Bundes- und Länderebene sowie den beteiligten Akteuren und Verbänden sicher. Für die Bearbeitung konkreter Themen und Fragestellungen bildet die NaLI Arbeitsgruppen, in die neben den NaLI-Mitgliedern auch weitere relevante Akteure, insbesondere Vertreterinnen und Vertreter der ärztlichen Berufsverbände und der Apothekerschaft, eingebunden sind.

Abschließend setzen sich alle NaLI-Mitglieder für eine transparente Aufklärung ein, um die Bevölkerung für das Thema Impfen zu sensibilisieren. Ein zentraler Bestandteil der Öffentlichkeitsarbeit der NaLI ist der Internetauftritt unter www.nali-impfen.de.

## Der Nationale Impfplan: Bestandsaufnahme der Umsetzung

Der Nationale Impfplan gibt seit über 10 Jahren wichtige Impulse zur Stärkung der Impfprävention. Sein Ziel war es, eine Übersicht über die Zuständigkeiten im föderalen Impfwesen in Deutschland zu schaffen und nationale Ziele zu formulieren. Diese orientieren sich an internationalen Vorgaben und sind in 6 Themenfelder gegliedert [2]:

1. Entwicklung und Zulassung von Impfstoffen,
2. Impfempfehlungen und Impfziele,
3. Umsetzung von Impfstrategien,
4. Information und Aufklärung,
5. unerwünschte Arzneimittelwirkungen,
6. Krankheits-Surveillance und Impfquoten-Erhebung.

Der Nationale Impfplan ist in 2 Teile unterteilt: Teil A enthält die Ziele des Impfwesens und beschreibt Maßnahmen zu deren Umsetzung. Teil B erläutert die Hintergründe, die zur Formulierung der Ziele geführt haben, und stellt die komplexe Organisation des deutschen Impfwesens transparent dar.

In den vergangenen 12 Jahren wurden viele der im Nationalen Impfplan festgelegten Ziele erfolgreich umgesetzt und weiterentwickelt. Zum Beispiel wurde mit der Gründung der NaLI im Jahr 2016 ein zentrales Element für die länderübergreifende Koordination von Impfthemen geschaffen (Teilziel I.1 „Organisatorische Voraussetzungen schaffen“). Die erstmals im Nationalen Impfplan bereitgestellte Übersicht über bestehende Aktivitäten im Impfwesen wurde in die NaLI-Website integriert und dort regelmäßig aktualisiert.

Neue gesetzliche Regelungen, wie das Präventionsgesetz, haben ebenso zur Verwirklichung der Ziele des Nationalen Impfplans beigetragen (z. B. Teilziel III.4 „Stärkung der Rolle des ÖGD“) wie auch die kontinuierliche Überprüfung und Anpassung der evidenzbasierten und transparenten Arbeitsmethode der STIKO mit Standardvorgehensweisen (SOP) zur Entwicklung von Impfempfehlungen (Teilziel II.1 „Hohe Transparenz und Evidenz der Impfempfehlungen der STIKO gewährleisten“). Der Nationale Impfplan hat dabei als wichtiger Anhaltspunkt und Impulsgeber für Entscheidungsträger auf Bundes- und Landesebene gedient. So konnten viele der im Nationalen Impfplan identifizierten strukturellen Impfhürden mittlerweile abgebaut werden.

Beispielsweise wurde das „fächerübergreifende“ Impfen ermöglicht (d. h., alle Ärztinnen und Ärzte, unabhängig von ihrer Fachrichtung, dürfen Schutzimpfungen durchführen), Rabattverträge zwischen Krankenkassen und Impfstoffherstellern wurden abgeschafft und Rechtsgrundlagen für neue Impf-Abrechnungsmöglichkeiten für den Öffentlichen Gesundheitsdienst (ÖGD) sowie die Betriebs- und Arbeitsmedizin geschaffen. Das niedrigschwellige Impfangebot wurde durch die Möglichkeit, in Apotheken impfen zu lassen, erweitert und der Impfschutz in medizinischen Einrichtungen durch die Regelungen in § 23a Infektionsschutzgesetz (IfSG) gefördert.

In Anlehnung an die Ziele der Weltgesundheitsorganisation (WHO) und vor dem Hintergrund der damaligen epidemiologischen Situation wurden auch spezifische Impfquotenziele in den Nationalen Impfplan von 2012 aufgenommen. Dazu gehören insbesondere Ziele für das Kindes- und Jugendalter, wie die Erhöhung der Impfquoten für die erste und zweite Masern-Mumps-Röteln-(MMR-)Impfung bei Kindern und Jugendlichen auf 95 % sowie die Steigerung der Inanspruchnahme von Auffrischungsimpfungen gegen Diphtherie, Tetanus, Pertussis und Polio bei Schulkindern oder Jugendlichen auf mindestens 90 %. Im Erwachsenenalter ist beispielsweise ein Ziel, die Influenza-Impfquoten bei Seniorinnen und Senioren und weiteren Risikogruppen auf über 75 % zu steigern [2]. Dank des Ausbaus der KV-Impfsurveillance am RKI^1^ (Auswertung von Abrechnungsdaten der kassenärztlichen Vereinigungen) können viele dieser Impfquotenziele inzwischen präziser evaluiert werden.

Obwohl bei nahezu allen empfohlenen Impfungen für Kinder und Jugendliche bis zum Beginn der COVID-19-Pandemie 2020 tendenziell steigende Impfquoten zu verzeichnen waren, sind einige Zielmarken, wie die 95 % bei der zweiten MMR-Impfung, noch nicht erreicht. Laut Auswertungen der KV-Impfsurveillance lag die Impfquote für die erste MMR-Impfung bei Kindern im Alter von 24 Monaten 2022 im bundesweiten Durchschnitt bei 93,7 %, für die zweite MMR-Impfung bei 80,5 %. Im Schuleintrittsalter wurde bereits 2020^2^ eine Impfquote von über 97 % für die erste MMR-Impfung erreicht, was die gesteckte Zielmarke erfüllte. Für die zweite MMR-Impfung lag die Impfquote jedoch noch darunter bei 93,2 % [5]. Im Gegensatz dazu bestehen bei Erwachsenen häufig noch größere Impflücken. Das von der WHO vorgegebene Influenza-Impfquotenziel von 75 % für Seniorinnen und Senioren sowie weitere Risikogruppen, das auch in den Nationalen Impfplan aufgenommen wurde, ist beispielsweise noch deutlich verfehlt. Laut Auswertungen der KV-Impfsurveillance lag die Impfquote in der Grippesaison 2020/2021 in den Indikationsgruppen bei 43,3 % der ab 60-Jährigen, 35,4 % der Personen mit impfrelevanten Grunderkrankungen und 17,5 % der Schwangeren [6]. Daten zu Auffrischimpfungen bei Schulkindern sind derzeit noch nicht bundesweit verfügbar, sondern nur in einzelnen Bundesländern – zum Beispiel in Bayern – anhand von Impfbuchdurchsichten der Gesundheitsämter [7, 8]. Welchen Einfluss die Pandemie auf die Inanspruchnahme einzelner Impfungen hatte, wird in aktuellen Auswertungen untersucht. Die bisherigen Daten deuten darauf hin, dass bei den Standardimpfungen von Kindern kein pandemiebedingter Einbruch zu verzeichnen ist [5, 9].

Auf der 8. Nationalen Impfkonferenz im Juni 2024 in Rostock-Warnemünde wurden diese und weitere Ergebnisse der Bestandsaufnahme zur Umsetzung der Ziele des Nationalen Impfplans auf einem Poster von Vertreterinnen und Vertretern der NaLI-Geschäftsstelle, des BMG sowie der Ausrichterländer der Nationalen Impfkonferenz vorgestellt [10, 11].

## Fortschreibung des Nationalen Impfplans

Wie bereits erläutert, gehört es neben der Erfolgskontrolle zu den wesentlichen Aufgaben der NaLI, den Nationalen Impfplan fortzuschreiben und weiterzuentwickeln. Dies umfasst sowohl die Aktualisierung bestehender Inhalte als auch die Aufnahme neuer Themen und Themengebiete sowie die Integration internationaler Konzepte und Strategien.

In den folgenden Abschnitten werden die bisherigen und laufenden Anstrengungen von Bund und Ländern bzw. der NaLI zur Fortführung des Nationalen Impfplans näher erläutert. In Infobox 1 ist die zeitliche Abfolge der Bestrebungen dargestellt.

### Überblick über das Impfwesen in Deutschland auf der NaLI-Website

Ein zentrales Anliegen von Bund und Ländern ist es, umfassende Informationen bereitzustellen, um Transparenz im Impfwesen für Fachleute und die interessierte Öffentlichkeit zu schaffen. Dies wurde bereits bei der Erstellung des Nationalen Impfplans berücksichtigt und durch die ausführlichen Darstellungen in Teil B umgesetzt [2].

Im Einklang mit ihrem Auftrag haben die NaLI und ihre Geschäftsstelle die bereits erwähnte Website www.nali-impfen.de erstellt, die im Mai 2019 online ging. Als zentrale Anlaufstelle zum Impfwesen bietet sie einen Überblick über Kampagnen und Regelungen in den Bundesländern und stellt die jeweiligen Verantwortlichkeiten dar. Darüber hinaus vermittelt sie durch ihre Kapitelstruktur die Schwerpunkte des Nationalen Impfplans sowie die Arbeit der NaLI. Durch die regelmäßige Überarbeitung der betreffenden Website-Kapitel bleiben die Informationen stets aktuell, was auch die fortlaufende Aktualisierung des Nationalen Impfplans zumindest teilweise gewährleistet. Dabei werden nicht nur bestehende Inhalte kontinuierlich angepasst, etwa wenn Änderungen der Gesetzeslage dies erfordern, sondern auch neue Inhalte erstellt, die nationale oder länderspezifische Impfthemen berücksichtigen [12].

Auch während der Corona-Pandemie wurden auf der NaLI-Website sukzessive neue Aspekte ergänzt. Zunächst wurden der aktuelle Stand der Impfstoffentwicklung, die Meldezahlen und später auch die STIKO-Empfehlung zur COVID-19-Impfung sowie deren Aktualisierungen hinzugefügt ebenso wie länderspezifische Informationen.

Die im 2‑Jahres-Rhythmus stattfindenden Nationalen Impfkonferenzen spielen als Forum für einen überregionalen Dialog aller Akteure im Impfwesen eine wichtige Rolle. Um dem Stellenwert dieser Konferenzen gerecht zu werden, bietet die NaLI-Website eine Übersicht über alle bisherigen Nationalen Impfkonferenzen. Zudem sind dort Berichtsbände verfügbar, die jeweils die Inhalte der Vorträge und Poster für eine spätere Einsichtnahme aufbereitet wiedergeben [13].

### Nationaler Aktionsplan 2015–2020 zur Elimination der Masern und Röteln in Deutschland

Deutschland hat sich zum Ziel der Europäischen Region der Weltgesundheitsorganisation (WHO-EURO) bekannt, Masern und Röteln zu eliminieren. Dieser Eliminierungsprozess wird in Deutschland von der „Nationalen Verifizierungskommission zur Elimination der Masern und Röteln“ (NAVKO) beim RKI begleitet. Die NAVKO bewertet die festgelegten Zielkriterien und Indikatoren und unterstützt Strategien, die zur Erreichung des Eliminationsziels sowie zur Überwachung der Fortschritte beitragen [14].

Zur Unterstützung dieses Prozesses wurde ergänzend zum Nationalen Impfplan der „Nationale Aktionsplan 2015–2020 zur Elimination der Masern und Röteln in Deutschland“ entwickelt. Expertinnen und Experten aus Bund und Ländern sowie ärztliche Verbände und weitere Institutionen erarbeiteten diesen Plan, der Ende 2015 – noch vor Gründung der NaLI – veröffentlicht wurde. Der Aktionsplan nimmt eine Bestandsaufnahme vor, formuliert nationale strategische und messbare Ziele und schlägt Maßnahmen sowie Aktionen zur Erreichung dieser Ziele vor [15].

Im Rahmen der Umsetzung des Nationalen Aktionsplans wurde zudem der „Generische Leitfaden für das Management von Masern- und Rötelnfällen und -ausbrüchen in Deutschland“ von der NaLI herausgegeben. Die darin enthaltenen Empfehlungen basieren unter anderem auf gesetzlichen Bestimmungen, bestehenden Empfehlungen einzelner Bundesländer und der WHO sowie auf Erfahrungen von Mitarbeitenden des öffentlichen Gesundheitsdienstes. Der Leitfaden wurde erstmals im Mai 2019 veröffentlicht und im Oktober 2020 in der zweiten Auflage aktualisiert [16].

### Nationales Konzept zur Förderung der Impfaufklärung und der HPV-Impfquoten

In den letzten Jahren wurden auf europäischer und internationaler Ebene weitere Impfpräventionsstrategien und -ziele formuliert, die im Nationalen Impfplan von 2012 noch nicht berücksichtigt oder nur am Rande abgebildet wurden. Ein Beispiel hierfür ist die Impfung gegen humane Papillomviren (HPV), die vor allem vor Gebärmutterhalskrebs (Zervixkarzinom) schützt, aber auch vor anderen Krebsarten im Anogenitalbereich sowie im Mund-Rachen-Raum.

Die WHO verabschiedete im August 2020 eine globale Strategie mit ehrgeizigen Zielen bis 2030, wie zum Beispiel eine 90 %-HPV-Impfquote bei 15-jährigen Mädchen weltweit, um Gebärmutterhalskrebs mittelfristig als Problem der öffentlichen Gesundheit zu eliminieren [17]. Auch der europäische Plan gegen den Krebs verfolgt das Ziel, die Zahl der HPV-bedingten Krebserkrankungen zu reduzieren [18]. Aufgrund ihres nachweislich positiven Effekts auf die Krankheitslast spielt die HPV-Impfung als primärpräventive Maßnahme dabei eine zentrale Rolle.

Bis 2021 stiegen die HPV-Impfquoten für eine vollständige Impfserie in Deutschland zwar sowohl bei Mädchen als auch bei Jungen größtenteils kontinuierlich an [19], sie blieben jedoch hinter den Erwartungen zurück, sodass das Präventionspotenzial nicht voll ausgeschöpft werden konnte. Aus diesem Grund forderte die GMK im Jahr 2021 alle Akteure im Gesundheitswesen auf, die Impfmotivation in der Bevölkerung zu stärken und insbesondere auf eine zeitgerechte Impfung hinzuwirken. In ihrem Beschluss wies die GMK darauf hin, dass bereits bestehende Aktivitäten in den Bundesländern als Vorbild dienen können, und regte an, dass die Akteure im föderalen Impfwesen ihre geplanten Maßnahmen der NaLI vorstellen und gegebenenfalls Rückmeldungen in die weitere Umsetzung sowie mögliche Synergien einfließen lassen [20].

Die NaLI nahm die Anregungen der GMK auf und beschloss daraufhin die Erarbeitung eines nationalen Konzepts zur Förderung der Impfaufklärung und der HPV-Impfquoten. Das HPV-Impfkonzept wird aktuell durch die NaLI-Arbeitsgruppe Masern/Röteln/HPV erarbeitet. Der damalige Stand des Konzepts wurde im Juni 2024 auf der 8. Nationalen Impfkonferenz in Rostock-Warnemünde vorgestellt [11].

Neben einer Übersicht und Bestandsaufnahme von Good-Practice-Modellen im föderalen Impfwesen soll das HPV-Impfkonzept der NaLI strategische Schwerpunkte und Vorschläge umfassen, die darauf abzielen, das Impfwissen zu verbessern und die Impfbereitschaft in Bezug auf HPV-assoziierte Erkrankungen sowie die HPV-Impfung zu erhöhen. Auch der Abbau struktureller Impfhürden, der niedrigschwellige Zugang zu Impfungen sowie digitale Lösungsansätze zur Erreichung der Zielgruppe sollen im Konzept berücksichtigt werden. Anhand bereits bestehender und bewährter Maßnahmen sollen konkrete Aktionen und Handlungsempfehlungen vorgeschlagen und mögliche Akteure zur Umsetzung benannt werden. Eine Evaluation der im Konzept formulierten Ziele ist für das Jahr 2030 vorgesehen [11].

Nach der Fertigstellung innerhalb der NaLI-Arbeitsgruppe wird das Konzeptpapier in der NaLI final abgestimmt und anschließend veröffentlicht. Das HPV-Impfkonzept ist – wie auch der Nationale Aktionsplan Masern/Röteln – Teil der Fortschreibung des Nationalen Impfplans. Um einen kontinuierlichen Überblick über aktuelle Aktionen und Maßnahmen zum Thema HPV-Impfung auch in Zukunft zu gewährleisten, wird das HPV-Impfkonzept auch auf der NaLI-Website unter dem Schwerpunkt HPV präsentiert [21]. Dies ermöglicht eine fortlaufende Ergänzung von Hinweisen und Links im Sinne eines dynamischen Konzepts.

### Nationaler Impfplan 2.0: Aktuelle Überarbeitung des Nationalen Impfplans von 2012

Der Nationale Impfplan von 2012 ist inzwischen in vielen Bereichen veraltet, insbesondere in der detaillierten Darstellung des deutschen Impfwesens in Teil B [2]. Wichtige Aktualisierungen hierzu werden seit 2019 auf der NaLI-Website veröffentlicht, jedoch decken diese nicht alle Aspekte des Nationalen Impfplans vollständig ab.

Zudem wurden viele der in Teil A des Nationalen Impfplans genannten Ziele in den letzten 12 Jahren erfolgreich umgesetzt und weiterentwickelt (siehe Abschnitt „Der Nationale Impfplan: Bestandsaufnahme der Umsetzung“; [10, 11]). Einige dieser Ziele wurden durch den Abbau von Impfhürden erreicht, während andere angesichts neuer Entwicklungen wie der Digitalisierung und neuer europäischer Impfziele einer erneuten Prüfung bedürfen. Das derzeit in Arbeit befindliche NaLI-HPV-Impfkonzept, das künftig Bestandteil des Nationalen Impfplans sein wird, orientiert sich bereits an neuen WHO-Zielen.

Vor diesem Hintergrund bedarf der Nationale Impfplan von 2012 einer umfassenden Überarbeitung. Mit dem Ziel, den Nationalen Impfplan zu aktualisieren und fortzuschreiben, gründete die NaLI im November 2024 eine neue Arbeitsgruppe. Diese Arbeitsgruppe wird sich auf die Prüfung und Anpassung der bestehenden Inhalte konzentrieren und ein zeitgemäßes Format entwickeln. Dabei soll auch die Ausrichtung an neuen internationalen Impfstrategien und neu formulierten Zielen auf europäischer Ebene berücksichtigt werden. Diese werden im folgenden Abschnitt näher vorgestellt.

## Internationale Ziele

Auf internationaler und europäischer Ebene wurden mehrere Impfziele, Impfstrategien und Empfehlungen von verschiedenen Akteuren formuliert, darunter die WHO, die EU und länderübergreifende Initiativen.

Im April 2020 publizierte die WHO mit ihren 194 Mitgliedstaaten die „Immunization Agenda 2030“ (IA2030) als Nachfolger des „Global Vaccine Action Plan 2011–2020“ [22, 23]. Zur Anpassung an regionale Gegebenheiten und Ziele wurde von der WHO Europa die darauf basierende „European Immunization Agenda 2030“ (EIA2030) mit Zustimmung aller Mitgliedstaaten, einschließlich Deutschlands, verabschiedet [24].

**Abb. 2:**
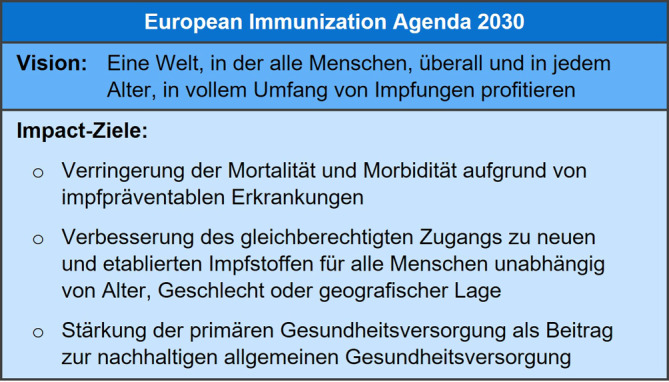
Vision und Ziele der European Immunization Agenda 2030 der WHO. (Quellen: Abbildung adaptiert an [11, 24]; Hinweis: Die dabei erfolgte Übersetzung wurde nicht von der Weltgesundheitsorganisation (WHO) angefertigt. Die WHO übernimmt keine Verantwortung für den Inhalt und die Richtigkeit der Übersetzung. Die englische Originalausgabe ist die verbindliche Fassung: [24])

Die EIA2030 vermittelt eine Vision und Strategie, um bis 2030 den vollen Nutzen von Impfungen innerhalb der WHO-Region Europa zu realisieren. Sie zielt darauf ab, eine national angepasste Umsetzung zu fördern, die den lokalen Kontext berücksichtigt. Die Hauptziele der EIA2030, auch als „Impact-Ziele“ bezeichnet, umfassen die Reduzierung von Todes- und Krankheitsfällen durch impfpräventable Erkrankungen, die Etablierung eines stärkeren Fokus auf „Equity“ – also dem gleichberechtigten Zugang zu Impfungen für alle („leave no one behind“) – sowie die Verbesserung der primären Gesundheitsversorgung in Bezug auf Impfungen (Abb. 2).

Zur Erreichung dieser Ziele definiert die EIA2030 7 strategische Schwerpunkte, die auf Kernprinzipien wie evidenzbasierten Entscheidungen und Empfehlungen beruhen. Diese Strategien sollen die Mitgliedstaaten bei der Implementierung und Umsetzung der EIA2030 unterstützen, um die darin festgelegten Ziele zu erreichen. Zudem umfasst die EIA2030 eine Strategie zur Überwachung und Bewertung des Fortschritts und der Zielerreichung. Zu diesem Zweck wurde eine Reihe von Indikatoren definiert [25], die regelmäßig über die Dauer der EIA2030 von der WHO für alle Mitgliedstaaten überprüft werden. Erst kürzlich hat die WHO Europa einen Zwischenbericht über den Fortschritt der EIA2030 zum Jahr 2023 veröffentlicht [26]. Zusätzlich wird im Rahmen der globalen IA2030 eine Übersicht der bislang verfügbaren Daten zur Erreichung der „Impact-Ziele“ angeboten, die die Darstellung für einzelne Länder weltweit ermöglicht. Diese Übersicht wird als Dashboard („Immunization Agenda 2030 Scorecard“) bereitgestellt [27].

**Abb. 3:**
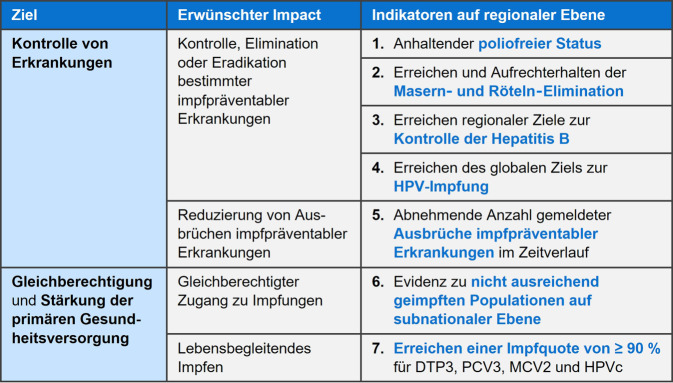
Impact-Indikatoren der „European Immunization Agenda 2030“ für alle Mitgliedstaaten. HPV, humane Papillomviren; DTP3: 3. Dosis eines Diphtherie/Tetanus/Pertussis-Impfstoffs; PCV3: 3. Dosis eines Pneumokokken-Konjugat-Impfstoffs; MCV2: 2. Dosis eines Masern-Impfstoffs; HPVc: vollständige Immunisierung mit einem HPV-Impfstoff. (Quelle: Abbildung modifiziert und übersetzt nach [24]; Hinweis: Die Übersetzung wurde nicht von der Weltgesundheitsorganisation (WHO) angefertigt. Die WHO übernimmt keine Verantwortung für den Inhalt und die Richtigkeit der Übersetzung. Die englische Originalausgabe ist die verbindliche Fassung: [24])

Für die Evaluierung der oben genannten Hauptziele der EIA2030 wurden Impact-Indikatoren definiert, welche eine regionale Bewertung ermöglichen (Abb. 3). Die Impact-Indikatoren 1 bis 5 betreffen das Hauptziel der Krankheitskontrolle von impfpräventablen Erkrankungen, wie beispielsweise die Bewahrung des poliofreien Status in ganz Europa, die Masern- und Röteln-Elimination, die Kontrolle von Hepatitis B (u. a. 90 % Impfquote für die 3. Dosis eines Hepatitis-B-Impfstoffs bei Kleinkindern), das Erreichen des globalen Ziels zur HPV-Impfung im Rahmen der WHO-Strategie zur Elimination des Gebärmutterhalskrebses (d. h. 90 % HPV-Impfquote bei 15-jährigen Mädchen) sowie die Reduzierung von Krankheitsausbrüchen. Die Impact-Indikatoren 6 und 7 betreffen den gleichberechtigten Zugang zu Impfungen und das lebensbegleitende Impfen. Letzteres wird definiert durch das Erreichen einer Zielimpfquote von mindestens 90 % für die jeweils 3. Dosis der Diphtherie‑/Tetanus‑/Pertussis- und Pneumokokken-Impfung im ersten Lebensjahr, die 2. Dosis der Masern-Impfung im zweiten Lebensjahr sowie eine vollständige HPV-Impfserie im Jugendalter (d. h. bei Mädchen im Alter von 15 Jahren). Weitere Impfquotenziele – wie eine 75 %-Influenza-Impfquote bei Seniorinnen und Senioren und medizinischem Personal – sowie die im Hinblick auf die angestrebte Elimination der Masern idealerweise zu erreichende 95 %-Impfquote für die 2. Masern-Impfung werden in den strategischen Schwerpunkten der EIA2030 zusätzlich erfasst [25].

## Fazit

Die komplexe Organisation des Impfwesens in Deutschland erfordert eine enge Abstimmung aller beteiligten Akteure. Gemeinsame Ziele wurden im Nationalen Impfplan von 2012 formuliert und die entsprechenden Maßnahmen zu deren Erreichung wurden festgelegt. Der Nationale Impfplan wurde seitdem durch verschiedene Maßnahmen weiterentwickelt und ergänzt. Einige der Ziele des Nationalen Impfplans wurden bereits erreicht, während andere aufgrund neuer Abläufe oder internationaler Zielvorgaben und Strategien überarbeitet oder neu aufgenommen werden müssen.

Seit Gründung der Nationalen Lenkungsgruppe Impfen (NaLI) im Jahr 2016 ist die Fortschreibung des Nationalen Impfplans eine ihrer zentralen Aufgaben. Für die gemeinsame Erarbeitung eines Nationalen Impfplans 2.0 plant die NaLI, die Grundsätze und Ziele der European Immunization Agenda 2030 (EIA2030) zu berücksichtigen.

### Infobox Nationaler Impfplan: Wichtige Bestrebungen und Fortführung

- *Nationaler Impfplan:* veröffentlicht 2012; erstellt durch Bund und Länder sowie weitere Expertinnen und Experten
- *Nationaler Aktionsplan 2015–2020 zur Elimination der Masern und Röteln in Deutschland:* veröffentlicht 2015; erstellt durch Bund und Länder sowie weitere Expertinnen und Experten
- *NaLI-Website* (www.nali-impfen.de) mit Darstellung des deutschen Impfwesens und wesentlicher Teile des Nationalen Impfplans seit 2019, kontinuierliche Aktualisierung der Inhalte durch die NaLI-Geschäftsstelle unter themenbezogener Mitwirkung der NaLI-Mitglieder und ständiger Gäste
- *Nationales Konzept zur Förderung der Impfaufklärung und der HPV-Impfquoten:* wird aktuell durch die NaLI-Arbeitsgruppe Masern/Röteln/HPV erarbeitet, die Veröffentlichung ist für 2025 geplant
- *Nationaler Impfplan 2.0:* Ende 2024 gründete die NaLI eine Arbeitsgruppe, die sich mit der Aktualisierung der Inhalte und einer zeitgemäßen Darstellung befasst

## Footnotes

1 https://www.rki.de/DE/Themen/Infektionskrankheiten/Impfen/Impfquoten/KV-Impfsurveillance/kvis_inhalt.html?templateQueryString=KV-Impfsurveillance. Zugegriffen am 20.01.2025.

2 Für Impfquoten im Vorschulalter lagen aufgrund der COVID-19-Pandemie zuletzt repräsentative Daten aus den Schuleingangsuntersuchungen 2020 vor.
